# Climate and health concerns of Montana’s public and environmental health professionals: a cross-sectional study

**DOI:** 10.1186/s12889-021-11737-1

**Published:** 2021-09-30

**Authors:** Lori Byron, Karen L. Akerlof

**Affiliations:** 1 Lori Byron, MS, MD, 2020 Alumna, Energy Policy and Climate Program, Johns Hopkins University, 3400 North Charles Street, Baltimore, MD 21218-2608 USA; 2 Department of Environmental Science and Policy, George Mason University, David King 3032, 4400 University Dr., MS 5F2, Fairfax, VA 22030 USA

**Keywords:** Climate change, Risk perception, Rural populations, Public health, Environmental health, Survey, Global warming

## Abstract

**Background:**

Rural health professionals stand at the forefront of community response to climate change, but few studies have assessed their perceptions of the threat. Further, no previous study has compared the opinions of environmental to public health professionals or extensively analyzed the factors related to these experts’ climate beliefs, risk perceptions, and issue prioritization.

**Methods:**

In conjunction with the Montana Climate Assessment’s 2021 *Special Report on Climate Change and Human Health,* the 479 members of the Montana Public Health Association and Montana Environmental Health Association were surveyed during September–October 2019, with 39% completing the survey. We summarized descriptive data about their perceptions of local climate-related changes and their beliefs that global warming is happening, is mostly human-caused, is a risk to human health, and that their offices and others should take action. We also evaluated which sociodemographic and risk perception factors related to these climate beliefs, risk perceptions, and workplace issue prioritization.

**Results:**

Health professionals in Montana, a politically conservative state, demonstrated high levels of awareness that global warming is happening, human-caused, and a threat to human health, well above reported rates of public concern. Eighty-eight percent said that global warming is occurring and 69% that it is mostly anthropogenic. Sixty-nine percent said that their own health was already affected by climate, and 86% said they were already seeing at least one climate change-related event in their communities. Seventy-two percent said that their departments should be preparing to deal with climate change’s health effects, but just 30% said that it is currently happening. We found no statistically significant differences between Montana environmental health and public health professionals in regression models predicting climate beliefs, risk perception, and prioritization. As in studies of the public, political ideology and the observation of local climate-related changes were the strongest factors.

**Conclusions:**

Montana environmental and public health officials said that departmental action was needed on climate change, indicating the readiness of rural health professionals to take action. Further studies of health professionals in rural regions are warranted.

**Supplementary Information:**

The online version contains supplementary material available at 10.1186/s12889-021-11737-1.

## Background

Anthropogenic climate change contributes to a wide array of increased human physical and mental health risks that manifest differently across communities due to varying hazards and vulnerabilities [1, 2]. While urbanization represents one of four global mega-trends tracked by the United Nations [3], much of the world’s population remains rural: 45% as of 2018 [4]. In the United States, rural areas constitute 97% of land area and 19% of the population [5]. These areas have distinct characteristics with implications for climate adaptation and health. Their economies are often closely tied to natural resources and agriculture with limited community capacity to adapt due to poverty and other social vulnerabilities [6, 7]. In the United States, rural communities are also less likely to be concerned about climate than those in urban areas [8]. Most of Montana’s population is rural (65%) [9]. In 2019, estimates placed 50 of the state’s 56 counties as less likely on average to say global warming is happening than the national average (67%) [8, 10].

Environmental and public health professionals recognize that environmental conditions affect human health [11, 12] and have called for action on climate change [13]. The Lancet Commission described tackling climate change as the greatest public health opportunity of the twenty-first century [14]. Because health is something that most people care about [15], communicating about the health effects of climate change can potentially help governments connect with wide audiences on the issue [16, 17]. Moreover, environmental and public health professionals, particularly nurses, are viewed as “trusted messengers” by the public; indeed, nurses have been the most trusted profession for the past 17 years in the Gallup Polls [18].

In this study we surveyed Montana environmental and public health professionals regarding their perceptions of climate change, its effect on human health, and the need for their offices and others to take action. Further, we analyzed how these perceptions relate to various factors, including sociodemographic and professional characteristics. To our knowledge, the climate risk perceptions of environmental and public health professionals have never been compared, nor have the climate change risk perceptions of health professionals in a frontier region of the United States like Montana been studied. Due to the importance of these professions for successful rural climate adaptation in their roles as content experts and trusted messengers [19], this study thus contributes a novel dimension to a growing literature on the role of health professionals in addressing climate change.

### Montana’s changing climate

Montanans are already experiencing impacts of climate change, such as fewer snow days and longer fire seasons [20]. We conducted this study of health professionals in association with Montana’s 2021 climate and health report: *Climate Change and Human Health in Montana: a Special Report of the Montana Climate Assessment, 2021* [19]. Released in December 2020, the report details current and projected health-related impacts of climate change in Montana based on increased temperatures, changes in precipitation patterns, altered ranges for infectious disease vectors, increased wildfires, and increased stress on crops (which can affect food supply, nutritional content, and cost of foods).

Montana also serves as home to a number of vulnerable populations who may be particularly affected by climate change [21]: rural residents, laborers in outdoor occupations, and indigenous communities. Montana is relatively sparsely populated with 6.8 people per square mile [22] compared to 92.9 persons per square mile nationally [23]. Outdoor occupations such as farming, fishing, forestry, construction, and extraction represent 65 out of every 1000 state jobs [24]. Indigenous residents—who like other racial and ethnic groups may be more socially vulnerable with fewer resources to adapt—comprise 6.6% of Montana’s population [22] versus 1.3% nationally [25].

### Climate change beliefs and risk perception

Climate change is frequently viewed by the lay public as abstract and distant in time and effect [26, 27]. People construe climate change as most likely happening elsewhere, to other people, or in the future. These characteristics of public risk perception can help explain why people choose not to engage in activities that might reduce the threat. Studies of risk perception suggest that perceived threat susceptibility and severity are important determinants of people’s behavioral responses [28, 29]. Hence it is notable that only 34% of Montanans in 2019 were likely to say that climate will harm them personally in the future, based on downscaled national survey estimates [10], compared to 42% nationally. (In 2020, Montana and U.S. percentages have increased, but the discrepancy remains; since this survey was conducted in 2019, 2019 data from Yale is cited.)

Public opinion data demonstrates consistently lower climate concern in Montana than nationally, typically by between 5 and 10 percentage points. The 2019 Yale Climate Opinion Maps estimate that 60% of Montana residents say that global warming is happening (67% nationally), while 45% say it is mostly human-caused (53% nationally), and 54% of Montanans are worried about it (60% nationally) [10]. Climate change perceptions have been well-documented as correlating with political affiliation, ideology, and worldviews [30]. Indeed, Montana is ranked as a “highly conservative” state according to 2018 Gallup data [31].

### Views of environmental and public health professionals on climate change

Public and environmental health professionals play an important role in climate adaptation [32] but typically have different organizational roles, expertise, and responsibilities for climate-related issues. For example, the Montana Public Health Association represents nurses, nutritionists, researchers, health educators, physicians and other licensed health practitioners (personal communication), while the Montana Environmental Health Association’s members include largely sanitarians, food inspectors, and disaster management personnel (personal communication). But few studies have assessed the climate change views of these officials [33]. Our academic literature search identified five U.S. studies of public health and/or environmental health professionals [34–38], three statewide studies [39–41] and three of physicians [42–44]. Other studies of health professionals’ views remain in the grey literature, such as surveys of Oregon officials [45] and members of the Association of State and Territorial Health Officers (ASTHO) [46]. Because researchers typically employ somewhat differently phrased survey questions and sampling strategies, comparisons are difficult, but in reviewing the 11 academic studies (Table 1; Additional Files 1, Supplementary Tables 1–2), we found:

- The majority of health professionals say that climate change is impacting their communities now with higher percentages saying they anticipate future impacts (Additional Files 1, Supplementary Tables 1–2);
- Identification of specific climate health impacts occurring in jurisdictions remains low, often at 50% or less (Table 1) with higher levels of concern for future climate health impacts than the present;
- Studies of National Environmental Health Association members [38, 48, 49] indicate somewhat lower levels of climate change concern than surveys with public health officials; and
- Few say that addressing climate change is a priority for their department (Additional Files 1, Supplementary Table 1).

**Table 1:** Surveys of U.S. public and environmental health professionals

	Authors / citation	Survey population	Study year	Current health impacts	Future health impacts
Public health	Maibach, Chadwick, McBride, Chuk, Ebi, & Balbus [34]	Dept directors (NACCHO)	2007–2008	12 impacts: 13% *unsafe sewage/septic-*56% *heat-related illness*	59% *jurisdiction will experience more serious health impacts in next 20 years*
	Roser-Renouf, Maibach, & Li [35]; NACCHO (report) [47]	Dept directors (NACCHO)	2011–2012	12 impacts: 11% *unsafe sewage/septic-*54% *heat-related illness*	61% *jurisdiction will experience more serious health impacts in next 20 years*
	Bedsworth [39]	Officers (California)	2007	–	94% *very/somewhat serious threat*; 9 impacts: 44% *food-borne illness*-91% *extreme weather*
	Polivka, Chaudry, & Crawford [36]	Dept nursing directors	2010	11 impacts: < 40% *malnutriton*-58% *vector-borne diseases*	65% *jurisdiction’s CC health impacts more serious within 20 years*
Public and environmental health	Carr, Sheffield, & Kinney [40]	Officials (New York)	2009	12 impacts: 25% *air quality-*50% *storms, hurricanes, floods*	39% *jurisdiction will experience more serious health impacts in next 20 years*
	Carter, Koman, Cameron, Ferguson, Jacuzzo, & Duvall [41]	Officials (Michigan)	2019	8 impacts: 12% *mental health-*53% *vector-borne disease*	8 impacts *increasing in next 20 years*: 21% *mental health-*66% *vector-borne disease*
Environmental health	Syal, Wilson, Crawford, & Lutz [37]	Dept directors	2010	12 impacts: 14% *malnutrition-*49% *air quality*	46% *jurisdiction’s CC health impacts serious*
	McAdams, Rehr, Kobayashi, & DeArman [38]; EcoAmerica & Lake Research Partners [48, 49]	Member survey (NEHA)	2016, 2017	5 cited personal impacts: 35% (2016), 33% (2017) *personally affected by breathing problems, such as asthma-*47% (2016), 46% (2017) *affected by summer heat waves*	3 cited impacts of concern (2017): 19% *health impacts from extreme weather;* 24% *vector-borne diseases;* 39% *health impacts from air pollution*

*NACCHO* National Association of County and City Health Officials; *NEHA* National Environmental Health Association.

Previous climate and health studies have surveyed environmental health as well as public health professionals, but no study to our knowledge has directly compared the two groups. In Syal and colleagues’ survey of environmental health directors in 2011 [37], only 46% said that the health effects of climate change in their jurisdiction would be serious. In surveys conducted with National Environmental Health Association members, a little more than a third of respondents (39%) were concerned in 2017 about the effects of increased asthma, allergies, and cardiorespiratory disease from higher rates of air pollution under climate change [48].

National surveys can also obscure large regional differences. A 2012 study of New York State local health department officials [40] —public and environmental health—found lower levels of concern and expertise than a national survey of members of the National Association of County and City Health Officials [35]. Less than a third (32%) of New York officials reported local effects from climate change already occurring in their jurisdiction and just 39% said that climate change posed a threat to public health in the next 20 years. Only one quarter of the respondents perceived climate change as an important priority for their local health department. In a 2009 study of local public health officers in California, Bedsworth [39] found much higher rates of concern and activity than in New York. A vast majority (94%) said that climate was a very or somewhat serious threat to public health; and majorities of the health departments reported programs in climate-related areas such as extreme heat, air pollution, and infectious disease.

Among the climate and health survey studies, only two to our knowledge have analyzed survey findings to establish the relationship between sociodemographic and professional characteristics, or other variables, on health professionals’ climate change perspectives. About half the professionals in Polivka’s public health nurses’ study in 2010 [36] said that their nursing division has a responsibility to address health-related effects of climate change, but most also said that they were not prepared to do so. A majority identified 4 out of 12 health effects as increased due to climate change with only subgroup differences by political ideology. However, there were differences on other measures by education, age, and political ideology. Less educated respondents were more likely to say that climate change is uncontrollable by humans than those with more years of college; younger respondents were more likely to say that nursing could lessen the health effects of climate change than those who were older; and liberals were more likely to say that climate change was anthropogenic and would have negative impacts than conservatives.

A 2011 study [37] assessed the relationship between environmental health directors’ environmental attitudes, political views, gender, and risk perception on implementation of climate adaptation programs in the department. The authors found that environmental attitudes and political views contributed to the risk perception of the directors; gender did not. Forty-nine percent said they felt a responsibility for their department to address the health effects of climate change. Moreover, environmental health directors’ climate and health risk perceptions accounted for 27% of the variance in the scope of climate change impacts addressed within programs.

Sociodemographic and other factors related to climate change beliefs have been better studied with the public. In a meta-analysis of the determinants of climate change beliefs using studies of members of the public from across 56 nations, sociodemographic factors—gender, age, income, education and race—were found to have little effect compared to political affiliation, values, trust in scientists, understanding of the climate science consensus, and experience of local weather change [30]. Van der Linden found similar relationships—though lesser effects from personal experiences—in a study of determinants of climate risk perceptions [50].

Therefore, the climate change beliefs, perceptions, and issue prioritization of health professionals in a rural, conservative state are likely to be affected by its political culture. At the same time, these experts have scientific training, actively engage with colleagues in medical and other scientific communities, and have direct experiences of changes in their community’s health [34, 35] that could also potentially influence their levels of issue concern. In order to further explore the risk perceptions of public and environmental health professionals, we pose the following research questions:

RQ1: What are the climate change local observations, beliefs, risk perceptions, and issue prioritization of public and environmental health professionals in Montana, a rural and conservative state?

RQ2: (a) What is the relationship between the professional and sociodemographic characteristics of rural health professionals and their climate change belief and risk perceptions? (b) What is the relationship between the professional and sociodemographic characteristics and climate risk perception of rural health professionals on their prioritization of climate change within their department? 

## Methods

We surveyed members of the Montana Public Health Association and Montana Environmental Health Association (MPHA/MEHA) between September 26, 2019 and October 30, 2019. MPHA has 379 members while MEHA is a smaller organization with just 100 members. The organizations’ members are widely geographically distributed across the state. At least one member of both MPHA and MEHA works in each of Montana’s 52 counties.

The 21-question survey was fielded both on paper and online. On average it took respondents just under 5 min to complete. At a joint MPHA/MEHA meeting in September 2019, members were given the option of completing the survey on paper versus waiting for an upcoming online survey link to be released the following week. The presidents of both organizations sent a link to the survey to their entire membership requesting their participation and notifying them that participants would be entered into a raffle for three $100 Amazon gift cards.

Regression analyses were completed in SPSS Statistics 27. Of 271 respondents, 47 were students and 39 did not complete two or more demographic questions, such as professional affiliation. Dropping these respondents from the dataset left a final sample of 185. Researchers have demonstrated that extreme weather events can influence climate change concern [51]. Of note, during 2019, there was a severe early snowstorm in September in Montana, but no severe wildfires in Montana [52, 53]. The survey received Johns Hopkins University IRB approval (Study #HIRB00009679).

### Survey measures

The questionnaire employed measures adapted from previous studies of health professionals and the public on climate change (see Table 1). The complete survey—including each measure’s wording—can be found in the Additional File 1. The questions address: 1) local climate change observations and assessments of current and future impacts; 2) climate change beliefs; 3) risk perception; and 4) prioritization of climate action by their offices and others. Demographics assessed in the survey included occupation, age, gender, geographic region, community size, political ideology, race, and ethnicity (See Supplementary Table 3, Additional File 1.) Political ideology was measured on a 1–9 scale with 9 being most conservative.

*Local climate change observations and assessments of current and future impacts.* Because of the politicization of climate change, the first set of measures did not employ either the term “climate change” or “global warming,” but instead asked whether respondents had observed changes in frequency of extreme heat days, late summer drought, flooding, forest fires, and extreme precipitation events in their community. The 2017 Montana Climate Assessment identified these as events that are occurring now and will increase over time in Montana [20]. For the purposes of the regression analyses, the number of reported changes that the professionals reported observing was summed. Respondents were also asked about current and future harm to their health and that of their patients from these impacts.

*Climate change beliefs.* In order to compare the results of the climate change public opinion questions to national- and state-level data from the Yale Project on Climate Change Communication [10], we asked whether *global warming* is happening (yes, no, don’t know) and its predominant cause (mostly human activity, mostly natural causes, it isn’t happening).

*Risk perception.* Respondents answered questions that asked them to judge whether climate change harms, benefits, or has no effect on human health at different temporal and social scales: 1) whether now or in the future; and 2) whether for yourself, your patients, in Montana, in the United States, or in other countries.

*Prioritization in addressing climate change.* A final set of questions asked the health professionals to relate how much of a prioritization climate change is—and should be—in their work and that of other professionals. Respondents provided their level of agreement, or disagreement, that the public health and environmental health effects of climate change should be a priority at their workplace (strongly agree—strongly disagree). They indicated whether at their workplace there has been any discussion or work around climate change, and suggested who should be addressing the causes and potential effects of climate change in Montana (businesses, elected officials, city/county governments, Montana state government, federal government, tribal governments, health care providers, public health officials, environmental health officials, individual citizens, non-profits).

## Results

After individuals with two or more missing demographic variables were dropped, the study response rate was 37% (MPHA) and 44% (MEHA). The professionals were mostly female (82%), white (94%), liberal (50%), with at least some college education (Table 2; Supplementary Table 3, Additional File 1). Respondents were geographically well-distributed across the state (Supplementary Fig. 1, Additional File 1). Half of the professionals serve in communities of 2000-50,000; another 40% work in communities of 50,000 or greater; 10% were in communities under 2500.

**Table 2:** Descriptive statistics for the sample

	*n*	Min	Max	*M*	*SD*
Age (18–44; 45–64; 65+)	185	1	3	1.62	0.64
Male	184	0	1	0.17	0.38
White/ Caucasian	185	0	1	0.94	0.24
Education	180	1	5	3.30	0.84
Political ideology	182	1	9	4.29	1.86
Environ health professional	185	0	1	0.24	0.43
Community size (< 2500; 2500-50,000; > 50,000)	184	1	3	2.30	0.64
Number of local observed climatic changes	179	0	5	2.63	1.65
Harm to patients	180	0	1	0.63	0.48
GW happening	185	0	1	0.88	0.32
GW human-caused	182	0	1	0.69	0.47
Harm to me	184	0	1	0.69	0.46
Priority for department	183	1	5	3.96	1.09

### Climate change local observations, beliefs, risk perceptions, and prioritizations of public and environmental health professionals in Montana [RQ1]

**Fig. 1:**
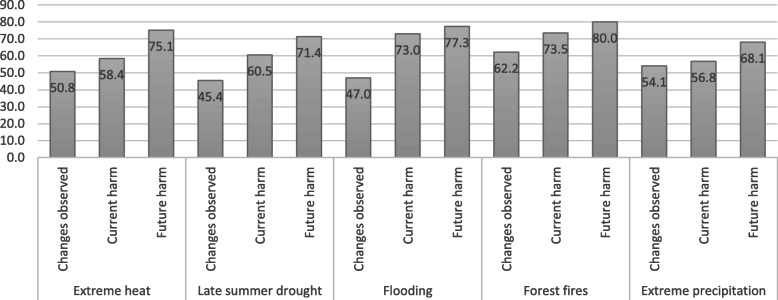
Montana health professionals’ perceptions of climate change-related events in their communities

*Local observations.* According to the 2017 Montana Climate Assessment [20], climate change is expected to increase extreme heat days, late summer drought, flooding, forest fire severity, and extreme precipitation events. The majority of respondents (86%) said that at least one of these phenomena are already occurring in their community. As shown in Fig. 1, about half of respondents (45 to 62%) said these events are already occurring, more than half (57 to 74%) said their community’s health is currently being harmed by each of these events, and an even higher number said their community’s health will be harmed more in the future (68 to 80%).

*Beliefs.* The vast majority of respondents (88%) said that global warming was happening, with 69% saying that it is mostly human caused.

*Risk perceptions.* Regarding perceived risk, 69% said that climate change was harming their personal health already, and 79% said that it would harm their personal health in the future.

*Prioritization.* While almost three out of four health professionals said that climate change should be a priority at their workplace (73%, somewhat agree or strongly agree), less than a third said there had been any discussion or work on the topic (30%). Almost all respondents (93%) said action is needed to address climate change; only 7% said no action is needed. Regarding who should take action on climate, 73% percent said that all of the entities listed should act on climate and 26% chose one or more: federal government (16%); environmental health officials (15%); Montana state government (14%); elected officials (11%); city/county governments (11%); tribal governments (9%); public health officials (8%); and individual citizens (8%); businesses (5%); non-profits (4%); and health care providers (2%), with the percentages showing the total for each individual entity.

### The relationship between professional and sociodemographic characteristics and climate change beliefs and risk perception [RQ2a]

Logistic regression models predicting survey participant selection of the responses that “global warming is happening” and that it is “mostly caused by humans” were both statistically significant (respectively, χ^2^(9) = 41.470, *p* < .001; χ^2^(9) = 31.345, *p* < .001) (Tables 3-4). The models correctly classified 89.1 and 74.6% of cases (0.415, 0.234, Nagelkerke *R*^*2*^). Of seven predictor variables only two were statistically significant in both the models: political ideology and community size (Tables 3-4). In both cases, increased conservatism was related to decreased likelihood of saying that global warming is happening and health experts serving communities between 2500 and 50,000 people—e.g., more rural areas—were less likely to say that global warming is happening. Occupation was not a significant predictor in either model.

**Table 3:** Logistic regression model predicting the response “global warming is happening”

	*B*	*SE*	Wald χ^2^	*df*	*p*	Odds ratio	95% C.I.for odds ratio	
							Lower	Upper
Age: 18–44 years^a^	−0.74	1.52	0.24	1	0.626	0.48	0.02	9.42
Age: 45–64 years^a^	−0.98	1.46	0.45	1	0.504	0.38	0.02	6.59
Male (Dichotomous)	1.28	1.16	1.21	1	0.270	3.58	0.37	34.68
Race: White/Caucasian (1); other (0)	0.12	1.20	0.01	1	0.919	1.13	0.11	11.91
Education (1–5)	−0.38	0.43	0.78	1	0.378	0.68	0.29	1.60
Political ideology (Conservatism ranked high, 1–9)	−0.93	0.26	12.51	1	0.000	0.40	0.24	0.66
Occupation: Environmental health (1); public health (0)	−0.72	1.00	0.52	1	0.471	0.49	0.07	3.46
Community size: Under 2500 people^b^	−0.11	1.29	0.01	1	0.933	0.90	0.07	11.18
Community size: 2500–50,00 people^b^	−1.95	0.97	4.09	1	0.043	0.14	0.02	0.94
Number of local observed climatic changes (0–5)	0.95	0.29	10.75	1	0.001	2.59	1.47	4.56
*Constant*	8.48	3.31	6.58	1	0.010	4839.60		

*n* = 170; ^a^Contrast category 65+ years; ^b^Contrast category over 50,000 people

**Table 4:** Logistic regression model predicting the response “global warming is caused mostly by human activity”

	*B*	*SE*	Wald χ^2^	*df*	*p*	Odds ratio	95% C.I. for odds ratio	
							Lower	Upper
Age: 18–44 years*	− 0.37	0.90	0.17	1	0.680	0.69	0.12	4.01
Age: 45–64 years*	−0.66	0.87	0.56	1	0.454	0.52	0.09	2.88
Male (Dichotomous)	0.47	0.62	0.57	1	0.449	1.59	0.48	5.32
Race: White/Caucasian (1); other (0)	0.05	0.78	0.00	1	0.947	1.05	0.23	4.84
Education (1–5)	−0.09	0.27	0.10	1	0.749	0.92	0.54	1.56
Political ideology (Conservatism ranked high, 1–9)	−0.46	0.13	13.36	1	0.000	0.63	0.49	0.81
Occupation: Environmental health (1); public health (0)	−0.22	0.53	0.17	1	0.679	0.80	0.29	2.26
Community size: Under 2500 people^b^	−0.76	0.75	1.03	1	0.310	0.47	0.11	2.03
Community size: 2500–50,00 people^b^	−0.82	0.45	3.22	1	0.073	0.44	0.18	1.08
Number of local observed climatic changes (0–5)	0.35	0.13	6.71	1	0.010	1.41	1.09	1.84
*Constant*	3.32	1.77	3.52	1	0.061	27.72		

*n* = 168; ^a^Contrast category 65+ years; ^b^Contrast category over 50,000 people

**Table 5:** Logistic regression model predicting the response “global warming harms … human health now for the people below [yourself]”

	*B*	SE	Wald χ^2^	*df*	*p*	Odds ratio	95% C.I. for odds ratio	
							Lower	Upper
Age: 18–44 years^a^	0.46	0.79	0.34	1	0.562	1.58	0.33	7.50
Age: 45–64 years^a^	0.10	0.78	0.02	1	0.896	1.11	0.24	5.13
Male (Dichotomous)	0.60	0.63	0.91	1	0.341	1.82	0.53	6.22
Race: White/Caucasian (1); other (0)	0.40	0.76	0.28	1	0.596	1.50	0.34	6.69
Education (1–5)	0.72	0.29	6.18	1	0.013	2.06	1.17	3.64
Political ideology (Conservatism ranked high, 1–9)	−0.34	0.12	7.42	1	0.006	0.71	0.56	0.91
Occupation: Environmental health (1); public health (0)	0.16	0.56	0.08	1	0.774	1.17	0.39	3.50
Community size: Under 2500 people^b^	0.71	0.77	0.86	1	0.355	2.04	0.45	9.17
Community size: 2500–50,00 people^b^	−0.23	0.46	0.25	1	0.616	0.80	0.32	1.95
Number of local observed climatic changes (0–5)	0.60	0.15	17.10	1	0.000	1.83	1.37	2.43
*Constant*	−2.14	1.73	1.53	1	0.216	0.12		

*n* = 170, ^a^Contrast category 65+ years; ^b^Contrast category over 50,000 people

The model predicting whether respondents say that climate change harms them now was also significant, χ^2^(10) = 55.364, *p* < .001. The model correctly classified 77.1% of cases (0.388, Nagelkerke *R*^*2*^). Of the eight predictor variables only three were statistically significant: education, political ideology, and number of observed local climate changes (Table 5). Higher education was related to an increased likelihood of saying that climate change harms them and the number of observed local climatic changes, while increased conservatism was associated with decreased likelihood of saying they were currently being harmed. Again, occupation was not a statistically significant predictor.

### The relationship between professional and sociodemographic characteristics and risk perception on climate change prioritization [RQ2b]

A linear regression model predicting respondents’ level of agreement that environmental and public health organizations should prioritize addressing the effects of climate change (Table 6) was significant *F*(11, 154) = 13.199, *p* < .001, accounting for 48.52% of the variation in the respondents’ agreement across the five-point measure (R^2^, 0.485). Of the nine predictors, four were significant. Men were more likely than women and other gender identifications to say that climate change should be a priority. So, too, were those who observed higher rates of local climate changes and those who said that their patients have been harmed from climate change. Conservatives were less likely to say that climate change should be a priority for their organizations.

**Table 6:** Regression model predicting agreement with “At my workplace, preparing to deal with the public health and environmental health effects of climate change should be a priority”

	*B*	SE	ß	*t*	*p*
*(Constant)*	4.32	0.59		7.33	0.000
Age: 18–44 years^a^	0.26	0.26	0.12	1.00	0.318
Age: 45–64 years^a^	0.04	0.26	0.02	0.15	0.881
Male (Dichotomous)	0.40	0.19	0.13	2.04	0.043
Race (White/Caucasian)	−0.05	0.28	−0.01	− 0.17	0.865
Education (1–5)	− 0.08	0.09	− 0.06	− 0.88	0.380
Political ideology (Conservatism ranked high, 1–9)	− 0.21	0.04	− 0.35	−5.10	0.000
Occupation: Environmental health (1); public health (0)	− 0.13	0.18	−0.05	− 0.72	0.475
Community size: Under 2500 people^b^	−0.19	0.25	−0.05	− 0.78	0.435
Community size: 2500–50,00 people^b^	− 0.27	0.14	− 0.12	−1.90	0.060
Number of local observed climatic changes (0–5)	0.16	0.04	0.24	3.62	0.000
Climate change harms my patients (Dichotomous)	0.59	0.15	0.26	3.80	0.000

*n* = 166; ^a^Contrast category 65+ years; ^b^Contrast category over 50,000 people

## Discussion

Public and environmental health professionals in a rural and conservative state demonstrate high levels of understanding that global warming is happening, human-caused, and has immediate health risks. Moreover, they say that their offices should prioritize this issue. However, few say that their offices are addressing the issue (30%). Like members of the public, factors such as political ideology and experiences of local change in their communities relate the most strongly to the experts’ responses.

### Comparison to other studies of health care professionals

In contrast to public concerns about climate change in Montana that typically rank lower than U.S. averages, health care professionals in Montana demonstrated similar or higher climate change concerns and perceived need for action compared to national studies of their colleagues. Most Montana health professionals said that global warming was happening (88%), mostly human caused (69%), and should be a priority at their workplace (73%). By way of comparison, one of the highest rates of climate concern and prioritization by health professionals was recorded in a 2014 survey of African American physicians in the National Medical Association (NMA) [42], where 61% were already seeing effects on health; and 75% said that physicians had a responsibility to address climate change with their patients. The relatively high rates of issue awareness and concern found in this study of Montana health professionals—roughly comparable to the 2014 NMA study—may be due in part to population-wide shifts in climate change beliefs during the decade and a half since these studies started [54].

Previous climate and health studies have surveyed environmental and public health professionals, but only two academically published surveys—of officials in Michigan and New York–included both groups within their sample [40, 41]. However, neither of those studies compared the two groups. While studies of National Environmental Health Association members indicated somewhat lower climate risk perceptions than public health professionals [38, 48, 49], our study found no difference in risk perception or climate prioritization between these two groups in Montana.

### Differences between health professionals and the public

Other research has shown that while health professionals can have very different views on climate change compared to the general public [35], they can also be subject to some of the same politically polarizing influences [34]. The present study illustrates both higher concern levels than the public and the influence of political ideology. In 2019, the Yale Climate Opinion Maps estimated that 60% of people in Montana were likely to say that global warming is happening [8, 10], as opposed this study’s finding of 88% of state public health professionals saying the same that year.

Public health professionals spend their careers addressing threats to the wellbeing of the people within their communities [36], so it might be inferred that they would be more informed about climate change and its health implications than the general public. But public and environmental health professionals, even in Montana, are distinctly different in political ideology and sociodemographic characteristics than state residents in general. As of 2018, 39% of Montanans said they were conservative, 38% moderate and 18% liberal [55]. In contrast, this sample of Montana health professionals was 23% conservative, 27% moderate, and 50% liberal. Additionally, 80% of the respondents were female [56].

### Factors related to professionals’ climate change beliefs, risk perceptions, and prioritization

Political ideology and local experiences of climate-related changes consistently significantly predicted responses that global warming is happening and mostly human-caused; both of these variables are also strongly correlated with public beliefs about climate change [30]. While local experiences of climate change may be motivated by either physical conditions or previously held beliefs [57], health experts might be expected to be more attuned to changes in conditions, especially as relates to community health. Interestingly, neither education nor expert status (environmental vs. public health) were consistently significant predictors, demonstrating little or no difference between environmental and public health experts, or differences between health professionals with some college experience versus those with advanced degrees. (Education was only a significant predictor of respondents who said that climate change was harming their health now.)

### Perceived need for climate action and what professionals can do

In 2018, at least half of registered U.S. voters--including Democrats, Independents, and liberal/moderate Republicans, but not conservative Republicans--said that citizens, the U.S. Congress, President Trump, their own member of Congress, and/or their local government officials should do more to address climate [58]. Most (73%) of the respondents in this study of Montana health professionals said that action on climate was needed by all of the above. For those who did not list “all,” federal government, environmental health, and state government officials ranked at the top of those the professionals said should be taking action, while health care providers fell at the bottom. The assumption that health care providers do not have a responsibility to address climate change is one that many in health care are attempting to change. Indeed, public and environmental health experts have many opportunities to get involved. Collaborations between local governments, community groups, and public health and environmental health professionals are needed, whether in writing and/or implementing a climate adaptation plan, educating the public on the hazards of extreme hear and the availability of cooling centers, or creating awareness campaigns on the dangers of smoke from forest fires and means of protection. By talking about climate, writing op-eds in the local newspapers, and educating legislators on its health hazards, they can help focus their communities on the decisions they face.

### Study limitations

This study found no significant difference in the beliefs and attitudes of environmental and public health professionals, a topic that had not been previously studied. But the number of these health professionals in Montana is relatively small: a larger U.S. study of public and environmental health professionals in other rural areas would be valuable. Although the survey response rate was relatively high with 39% fully completing the survey, we do not have demographic information for the two member organizations to fully assess the representativeness of the sample. The online survey was advertised by the MEHA and MPHA leadership with no mention climate or global warming, but the 17% of respondents who participated in a paper version of the study at MPHA’s annual meeting met the lead author and may have been aware that the study was about climate change, leading to response bias.

## Conclusion

Montana’s health professionals are already aware of climate change’s risks and want to see their offices and others more actively engage on the issue. Because of the level of expertise and community knowledge held by these professionals, information about their concerns may be helpful for the public and policymakers, much as climate organizations have spotlighted faith groups as opinion leaders [59]. The positions of trust that these experts hold in their communities potentially make them ideally situated to lead discussions on how to address climate change in rural areas. As health professionals become more aware that a large number of them– even in rural conservative states – are concerned, it may potentially open up spaces for wider conversations with their colleagues and patients.

## Supplementary Information

**Additional file 1.**

**Additional file 2.**

## Data Availability

The survey tool, supplementary Tables 1,2, ands 3, and a heat map is located in Additional File 1. The dataset supporting the conclusions of this article is available in an SPSS file in Additional File 2.

## Abbreviations

APHA: American public health association
EH: Environmental health
IRB: Institutional review board
MEHA: Montana environmental health association
MPHA: Montana public health association
NEHA: National environmental health association
NMA: National medical association
PH: Public health
US: United States
