# Supplementary material for: Climate and health concerns of Montana’s public and environmental health professionals: a cross-sectional study
Source: BMC Public Health. 2021 Sep 30;21:1778. doi: 10.1186/s12889-021-11737-1 (PMC8485501; doi:10.1186/s12889-021-11737-1)
Supplement: Supplementary file 1 — Additional file 1. [file 12889_2021_11737_MOESM1_ESM.docx]

**ADDITIONAL FILE # 1:**

**SUPPLEMENTARY MATERIALS**

**Supplementary Materials: Part A**

**SupplementaryTable 1.** Since 2007, researchers have conducted surveys with public and environmental health professionals on their perceptions of climate impacts.

|  | **Authors / citation** | **Survey population** | **Study year** | **Current climate impacts** | **Future climate impacts** | **Dept issue prioritization** |
| --- | --- | --- | --- | --- | --- | --- |
| Public health | Maibach, Chadwick, McBride, Chuk, Ebi, & Balbus (34) | Dept directors (NACCHO) | 2007- 2008 | 69% *jurisdiction experienced CC in the last 20 years* | 78% *jurisdiction will experience CC in the next 20 years* | 51% *important priority for my health dept* |
|  | Roser-Renouf, Maibach, & Li (35); NACCHO (report) (49) | Dept directors (NACCHO) | 2011- 2012 | 66% *jurisdiction experienced CC in the last 20 years* | 77% *jurisdiction will experience CC in the next 20 years* | 41% *important priority for my health dept* |
|  | Bedsworth (39) | Officers (California) | 2007 | − | − | − |
|  | Polivka, Chaudry, & Crawford (36) | Dept nursing directors | 2010 | − | − | − |
| Public and environmental health | Carr, Sheffield, & Kinney (40) | Officials (New York) | 2009 | 32% *jurisdiction experienced CC in the last 20 years* | 57% *jurisdiction will experience CC in the next 20 years* | 25% *important priority for my health dept* |
|  | Carter, Koman, Cameron, Ferguson, Jacuzzo, & Duvall (41) | Officials (Michigan) | 2019 | 62% *jurisdiction experienced CC in the last 20 years* | 76% *jurisdiction will experience CC in the next 20 years* | 35% *priority for my health dept* |
| Environmental health | Syal, Wilson, Crawford, & Lutz (37) | Dept directors | 2010 | − | − | − |
|  | McAdams, Rehr, Kobayashi, & DeArman (38); EcoAmerica & Lake Research Partners (47,48) | Member survey (NEHA) | 2016, 2017 | 65% (2016), 70% (2017) *have noticed changes in weather where they live* | − | − |
|  |  |  |  |  |  |  |
|  | *NACCHO =National Association of County and City Health Officials; NEHA = National Environmental Health Association* | | | | | |

**Supplementary Table 2.** Three studies have surveyed physicians.

| **Authors / citation** | **Survey population** | **Study year** | **Current climate impacts** | **Current health impacts** | **Future health impacts** |
| --- | --- | --- | --- | --- | --- |
| Sarfaty, Bloodhart, Ewart, Thurston, Balmes, Guidotti, & Maibach (43) | Member survey (ATS) | 2014 | 81% *personal experience of CC impacts to some extent* | 6 impacts: 26% *(food/water-borne illness diarrhea)* - 77% (air pollution related illness severity) | 6 impacts (next 10-20 years): 48% *(food/water-borne illness diarrhea)* - 80% *(air pollution related illness severity)* |
| Sarfaty, Mitchell, Bloodhart, & Maibach (42) | African American physicians (NMA) | 2014 | 48% *personal experience of CC impacts great deal/moderate amount*; 66% *CC has harmed people in area a great deal / moderate amount* | 61% *CC affecting patient health a great deal / moderate amount;* 7 impacts: 40% *(mental health)* - 88% *(injuries from severe weather)* | 6 impacts (next 10-20 years): 67% *(food/water-borne illness diarrhea)* - 91% *(air pollution related illness severity)* |
| Sarfaty, Kreslake, Casale, & Maibach (44) | Member survey (AAAAI) | 2015 | 73% *personal experience of CC impacts to some extent* | 6 impacts: 23% *(food/water-borne illness diarrhea)* - 73% *(air pollution related illness severity)* | 6 impacts (next 10-20 years): 38% *(food/water-borne illness diarrhea)* - 72% *(air pollution related illness severity)* |
|  |  |  |  |  |  |

*NMA = National Medical Association; ATS = American Thoracic Society; AAAAI = American Academy of Allergy Asthma and Immunology*

**Supplementary Table 3.** Sample demographic characteristics

| *n*=185; percentages may not sum  to 100 due to rounding | | **Public Health Respondents (%)** | **Environmental**  **Health**  **Respondents (%)** |
| --- | --- | --- | --- |
| **Gender** | Female  Male  Other | 89  10  1 | 59  39  2 |
| **Education** | High School  Some college  Bachelor’s Degree  Master’s Degree  Doctor’s Degree | 1  20  43  27  10 | 0  0  60  38  2 |
| **Occupation**  *(Respondents may select one or more)* | ***Environmental Health***  Sanitarian  ***Public Health***  RN  WIC  Dietician  Researcher  PA, NP, MD  Admin  Retired  Other | 0  32  6  1  7  7  23  2  40 | 100  0  0  0  0  0  7  2  16 |
| **Age** | 18-44  45-64  65-over | 52  39  9 | 32  61  7 |
| **Race and**  **ethnicity** | American Indian  Asian  Latino or Hispanic  Black/African-American  Native Hawaiian/ Pacific Islander  White/Caucasian  Other | 5  2  1  0  1  94  1 | 0  2  0  0  0  93  5 |
| **Community population size** | Under 2,000  2,000-50,000  Over 50,000 | 12  54  34 | 2  36  61 |
| **Political**  **Ideology** | Conservative  Centrist  Liberal | 22  26  52 | 27  29  43 |

**Supplementary Figure 1.** A heat map demonstrates the distribution of survey respondents. Montana’s Eastern and Northeastern regions are the least populated.


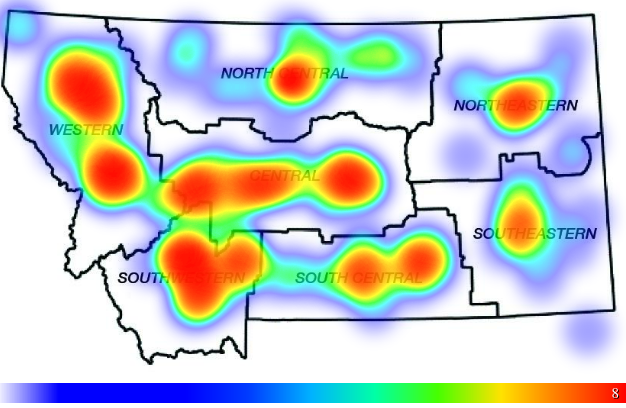


**Supplementary Materials Part B: Survey**

I am requesting your help in an important study about public and environmental health in Montana.  Your expertise as leaders in public health in your communities in Montana is valued by many.  By taking this time to share your thoughts, you will help us understand how to better develop future health services for Montanans, such as programs that assist communities during forest fires,  heat waves, and other extreme weather events, with a long range goal of supporting broader public and environmental health measures in Montana to address these health risks.  This topic has been highly researched elsewhere but never in Montana. This survey is for my Masters’ Thesis from Johns Hopkins University and is done in collaboration with researchers from Montana State University.  Please contact me with any questions at lori.byron@gmail.com 
This survey is anonymous unless you choose to share identifiers to receive information at the end of the survey.
By completing this survey or questionnaire, you are consenting to be in this research study.  Your participation is voluntary and you can stop at any time.

- I have read this form and agree to participate in this study.
- I do not wish to participate in this study. {Skip To End of Survey}

1. **Career reasoning   What was the main reason you chose your current occupation?  (choose one)**

- To help people/community
- It is an influential position in my community
- To study in a field I loved
- To be in the community where I now am
- To protect our environment
- It is a good way to make a living
- Other (please fill in) ________________________________________________

1. **Would you say there has been a change over time in the frequency of these events in your community (the population you serve in your professional position)?**

|  | Yes | No | Not sure |
| --- | --- | --- | --- |
| Extreme Heat Days |  |  |  |
| Late Summer Drought |  |  |  |
| Flooding |  |  |  |
| Forest Fires |  |  |  |
| Extreme Precipitation Events (microbursts, storms, etc.) |  |  |  |

1. **Do the following types of events currently harm the public or environmental health in your community?**

|  | Yes | No | Don't know |
| --- | --- | --- | --- |
| Extreme Heat Days |  |  |  |
| Late Summer Drought |  |  |  |
| Flooding |  |  |  |
| Forest Fires |  |  |  |
| Extreme Precipitation Events |  |  |  |
| Food/Water Borne Illness |  |  |  |
| Vector Borne Illness |  |  |  |

1. **Do you anticipate that the following types of events will harm the health of your community in the future?**

|  | Yes | No | Don't know |
| --- | --- | --- | --- |
| Extreme Heat Days |  |  |  |
| Late Summer Drought |  |  |  |
| Flooding |  |  |  |
| Forest Fires |  |  |  |
| Extreme Precipitation Events |  |  |  |
| Food/Water Borne Illness |  |  |  |
| Vector Borne Illness |  |  |  |

*Over the last 50 years, Montana scientists have observed changes in Montana’s climate (for example, the 2017 Montana Climate Assessment). We would like to hear from public health leaders like you about potential health effects of these changes.*

1. **Global warming refers to the idea that the world’s average temperature has been increasing over the past 150 years, may be increasing more in the future, and that the world’s climate can change as a result.** 
   What do you think: Do you think that global warming is happening?

___Yes ___No ___Don't Know

1. **Assuming global warming is happening, do you think it is...**

- Caused mostly by human activity
- Caused mostly by natural causes
- Neither, because it isn't happening
- Other: ________________________________________________

1. **Do you think climate change harms, benefits, or has no effect on human health now for the people below ?**

|  | Harms | No Impact | Benefits | Don't know |
| --- | --- | --- | --- | --- |
| In other countries |  |  |  |  |
| In the United States |  |  |  |  |
| in Montana |  |  |  |  |
| For your patients |  |  |  |  |
| For yourself |  |  |  |  |

1. **Do you think climate change harms, benefits, or has no effect on human health in the future for the people below?**

|  | Harms | No impact | Benefits | Don't know |
| --- | --- | --- | --- | --- |
| in other countries |  |  |  |  |
| in the United States |  |  |  |  |
| in Montana |  |  |  |  |
| For your patients |  |  |  |  |
| For yourself |  |  |  |  |

1. **Are you concerned about the effects of climate change-related mental health problems in your community, either now or in the future? (check all that apply)**

- At present, yes
- At present, no
- In the future, yes
- In the future, no
- Not sure

1. **At my workplace, preparing to deal with the public health and environmental health effects of climate change should be a priority**

- Strongly agree
- Somewhat agree
- Neither agree nor disagree
- Somewhat disagree
- Strongly disagree

1. **At your workplace have there been any discussion, or work, around climate change?**

___Yes, Details: ______ ___No

1. **In Montana, who should be working to address the causes and potential effects of climate change? (check all that apply)**

- No one/Not needed
- Other ________________________________________________
- ALL OF THE BELOW
- Businesses
- Elected Officials
- City/County Governments
- Montana State Government
- Federal Government
- Tribal Governments
- Health Care Providers
- Public Health Officials
- Environmental Health Officials
- Individual Citizens
- Non-profits

1. **On this map, what region are you in? (use your best guess if you think you are near a border between these divisions) Click on your region.**


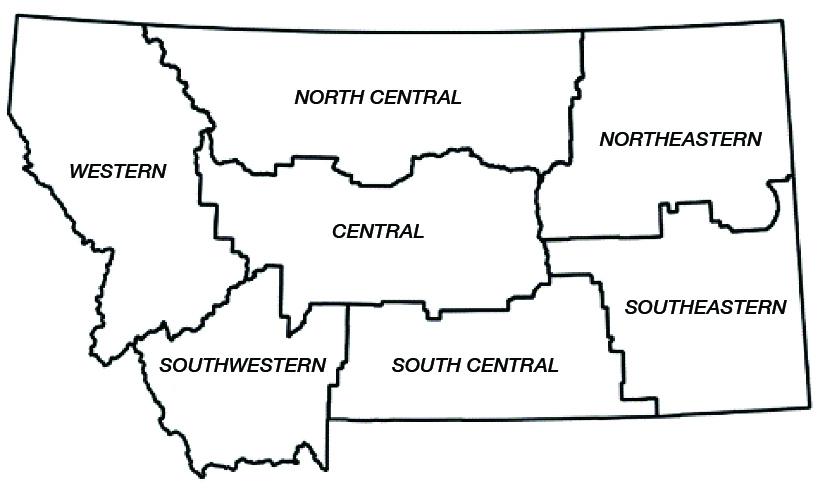


1. **Please check the highest degree you have earned. (check one)**

- High School
- Some college education
- Bachelor's Degree
- Master's Degree
- Doctorate

1. **Please check your current occupation (check all that apply)**

- RN
- WIC employee
- Registered Dietician
- Sanitarian
- Researcher
- PA/NP/APRN
- Physician
- Administraton
- Other, or clarification:______
- Retired, past career:________
- Student, intended career:_____________

1. **Please check your gender:**

___Male ___Female ___Prefer not to answer ___Other: ______

1. **Please check your age group:**

____18 - 44 ____45-64 ____65 and older

1. **Please check your race and/or ethnicity (check all that apply)**

- American Indian or Alaska Native
- Asian
- Black or African American
- Latino or Hispanic
- Native Hawaiian or Pacific Islander
- White or Caucasian
- Other: ________________________________________________

1. **What is the size of the population of your community? (The population that you serve in your professional capacity)**

___Under 2500 ___2500-50,000 ___Over 50,000

1. **Would you like to receive (check all that apply):**

- a link to the Health Section of the Montana Climate Assessment when it becomes available
- information on climate and health
- patient information - brochures - on climate and health
- slide decks on climate and health
- information on the Montana Health Professionals for a Healthy Climate
- other information: ________________________________________________

PLEASE PROVIDE YOUR EMAIL TO RECEIVE INFORMATION:

1. **Where would you place yourself on a scale of 1 to 9**

- 1 (most liberal)
- 2
- 3
- 4
- 5
- 6
- 7
- 8
- 9 (most conservative)
